# Low number of luminance levels in the luminance noise increases color discrimination thresholds estimated with pseudoisochromatic stimuli

**DOI:** 10.3389/fpsyg.2014.01291

**Published:** 2014-12-23

**Authors:** Givago S. Souza, Felecia L. Malone, Teera L. Crawford, Letícia Miquilini, Raílson C. Salomão, Diego L. Guimarães, Dora F. Ventura, Malinda E. C. Fitzgerald, Luiz Carlos L. Silveira

**Affiliations:** ^1^Instituto de Ciências Biológicas, Universidade Federal do ParáBelém, Brazil; ^2^Núcleo de Medicina Tropical, Universidade Federal do ParáBelém, Brazil; ^3^Department of Biology, University of MemphisMemphis, TN, USA; ^4^College of Medicine, University of Tennessee Health Science CenterMemphis, TN, USA; ^5^Instituto de Psicologia, Universidade de São PauloSão Paulo, Brazil; ^6^Department of Anatomy and Neurobiology, University of Tennessee Health Science CenterMemphis, TN, USA

**Keywords:** pseudoisochromatic stimulus, compound stimulus, color-luminance interaction, Cambridge Color Test, color vision, P pathway

## Abstract

In pseudoisochromatic stimuli the presence of spatial and luminance noise forces the subject to discriminate the target from the background solely on the basis of chromaticity difference. Color-blind subjects may show difficulty to identify the target due to the elimination of borders and brightness clues caused by the luminance and spatial noise. Few studies have fully described the features of pseudoisochromatic stimuli. Fewer investigators have focused their studies in the effects of specific pseudoisochromatic parameters on color discrimination. We used the Cambridge Color Test (CCT) to investigate the influence on color discrimination thresholds due to the number of luminance levels present in the luminance noise. The CCT default has six luminance steps; however, in our investigation a total of eight different conditions were tested from 2 to 16 luminance steps. It was found that the CCT provided very robust values for color discrimination thresholds, which were degraded only for very small number of luminance steps. When the number of steps was increased, the color discrimination thresholds improved from 2 to 6 luminance steps and gradually reached a plateau for 10 or more luminance steps. The area of color discrimination ellipses as a function of luminance steps matches the relative proportion of ineffective contrasts between mosaic patches as a function of luminance steps, assuming that contrast becomes ineffective for values 18.6% or less. The lower number of color and luminance interactions in these conditions could explain the measured increase of color discrimination thresholds. The primary conclusion from this investigation was that results from pseudoisochromatic tests should have their parameters described in more detail. This type of description would allow a better understanding of the results provided, interpretations, and therefore cross study comparison of results obtained from different laboratories.

## INTRODUCTION

Deficiencies in color vision decrease the ability to discriminate certain colors under specific circumstances. The inability to discriminate colors can result in visual problems in daily life. Full characterization of color vision deficiency would allow subjects with decreased color discrimination to potentially conduct necessary adjustments to their visual deficiencies and live a more normal life. Testing for color vision deficiencies may identify the existence, type, and severity of defects, providing a basis for the evaluation of the defect’s impact on personal and professional performance (Dain, 2004).

Multiple types of visual testing exist that are used to measure the level of color perception, including pseudoisochromatic plate tests (Birch, 2001). Pseudoisochromatic plates employ targets broken into patches of a given chromaticity embedded in a background of patches of different chromaticity, but the two sets of patches – those that compose the target and those that compose the background – vary in size and luminance, to isolate and measure the subject’s color discrimination performance (Mollon, 2003).

The pseudoisochromatic tests were developed based on the suggestions of Jakob Stilling (1842–1915) to eliminate the edges between target and background by breaking the stimulus into a mosaic with patches of different sizes (spatial noise) and brightness (luminance noise). One major aspect of pseudoisochromatic stimuli is that the presence of spatial and luminance noise requires the subject to heavily rely on chromatic signals to differentiate the target from its background (Mollon and Reffin, 1989; Regan et al., 1994).

The first pseudoisochromatic test to become largely used, named for Shinobu Ishihara (1879–1963), was introduced in the early 1900s to identify deficiencies in red–green color vision (Heidary and Gharebaghi, 2013). Although the Ishihara test is still widely used, it failed to properly categorize many defects of color vision, especially those of tritan category (Aarnisalo, 1979). The American Optical Hardy-Rand-Rittler (AOHRR) test was produced in several versions during the mid and late 1900s due to issues with the saturation of red and green plates. This test distinguished between protans and deutans with difficulties, because it did not contain a sufficiently large range of weak and strong stimuli to correctly identify the specific color vision defect (Walls, 1959). The Standard Pseudoisochromatic Plates (SPP) test is presented either in a version for congenital visual defects (SPP-C) or another version for acquired visual defects (SPP-A), but it is more affected by the duration per test item and viewing distance than other pseudoisochromatic tests such as the Ishiara test and the City University Color Vision Test (CUCVT; Somerfield et al., 1989; Dain, 2004).

Although there is wide use of pseudoisochromatic stimuli in color vision investigation, few studies have focused on how the features of the stimuli themselves could influence the visual perception. Most studies focused on the test conditions such as illuminance of the stimulus plates, viewing distance, and exposure time (Long et al., 1984; Somerfield et al., 1989). These conditions have been found to significantly affect individual performance on visual screening tests. The administration of multiple and/or different plate tests may require viewing conditions within certain standards in order to ensure test validity and comparability (Long et al., 1985). It has been observed that patients with low visual acuity had a high rate of recognition with utilization of the Ishihara plates in color discrimination tests (Gordon and Field, 1978). These authors suggested that the elimination of high spatial frequency information, by the low visual acuity, might explain the better performance of the subjects (Gordon and Field, 1978). Taylor and Woodhouse showed that blurring could also improve the recognition and therefore discrimination performance using pseudoisochromatic plates in deutans (Taylor and Woodhouse, 1979).

Examples of studies that investigated how specific features of the pseudoisochromatic stimuli could potentially influence visual perception were those that provided the basis for the development of Cambridge Color Test (CCT; Mollon and Reffin, 1989; Reffin et al., 1991; Regan et al., 1994). Mollon and Reffin (1989) used pseudoisochromatic stimuli and modulated the target chromaticity along several axes of the chromaticity diagram using a staircase method. The procedure allowed them to estimate several color discrimination thresholds around a given chromaticity locus and to plot the corresponding MacAdam ellipse. They observed that color discrimination ellipses of trichromats and dichromats corresponded well to the color vision genotype of the subjects. Normal data for color discrimination using the CCT have been published by Ventura et al. (2003), Paramei (2012), and Paramei and Oakley (2014). Other investigatiors have applied similar paradigms to investigate color discrimination in both children and non-human primates (Mancuso et al., 2006; Goulart et al., 2008, 2013).

The amount of luminance noise represents an important parameter to characterize a pseudoisochromatic stimulus. Changes in the composition of the luminance noise might influence the visual perception of the target, because it can change the interaction of luminance and chromatic information in the visual scene (Switkes et al., 1988; Ingling and Grigsby, 1990; Logothetis et al., 1990; Gur and Akri, 1992; Clery et al., 2013). In the current study we investigated how the number of luminance levels in the luminance noise of pseudoisochromatic stimuli influenced the color discrimination ellipses.

## MATERIALS AND METHODS

### SUBJECTS

Nine subjects (25.67 ± 3.24 years old) were included in the current study. All subjects gave written consent to participate in the study. This study agreed with the tenets of the Declaration of Helsinki and it was approved by the Ethical Committee for Research in Humans, Tropical Medicine Nucleus, Federal University of Pará (Report #570.434) and the IRB at UTHSC. None of subjects had any history of ophthalmological, neurological, or systemic diseases that could affect visual performance. Verification of visual function was performed by an ophthalmologist that conducted the following initial examinations: ophthalmoscopic and retinoscopic exam, slit lamp exam of the eye media, refractive state measurement, Snellen visual acuity test, and Ishihara plate test. All subjects were monocularly tested and the eye with the highest Snellen visual acuity, based on prior initial ophthalmological examination, was the eye used for the pseudoisochromatic stimuli examination. All the subjects were normal regarding the results of ophthalmological exam, had normal or corrected to 20/20 visual acuity, and performed with no mistakes in the Ishihara’s plate test.

### STIMULATION

The stimuli were generated in a ViSaGe system (Cambridge Research System, CRS, Rochester, England, UK) and exhibited in a 21^′′^ CRT display with high spatial, temporal, and chromatic resolution (1600 × 1200 pixels, 125 Hz, 14 bits, Mitsubishi, Tokyo, Japan). Luminance and chromaticity were measured and gamma-correction was performed to calibrate the monitor using a colorimeter ColorCal (CRS).

We used the CCT software (CCT, CRS) to estimate color discrimination ellipses around coordinates (*u*′ = 0.1977, *v*′ = 0.4689) of the CIE 1976 Color Space. Each stimulus was comprised of an assortment of discrete circular patches with their own random size and luminance. The minimum and maximum luminance values of the luminance noise were 8 and 18 cd/m^2^, respectively. Embedded in this field of spatial and luminance noise there was a target with the shape of a Landolt’s “C” formed by its own assortment of patches differing in chromaticity from those of the background (Regan et al., 1994). Subjects were placed 3.25 meters away from the monitor in a dark room. At this distance, the Landolt’s “C” gap, outer diameter, and inner diameter measured 1, 4.3, and 2.2∘ of visual angle, respectively. The stimulus was shown for 3 sec. The target chromaticity was modulated along eight chromatic vectors radiating from the background chromaticity.

### PSYCHOPHYSICAL PROCEDURES

The CCT uses a four-alternative forced choice staircase to estimate color discrimination thresholds along each chromatic vector. The subject’s task was to identify the orientation of the Landolt’s “C” gap (up, down, left, or right). The subject’s response was recorded using a four-button response box (CB6, CRS). Each correct response resulted in a decrease of the chromatic vector and an error resulted in an increase of the chromatic vector.

**FIGURE 1:**
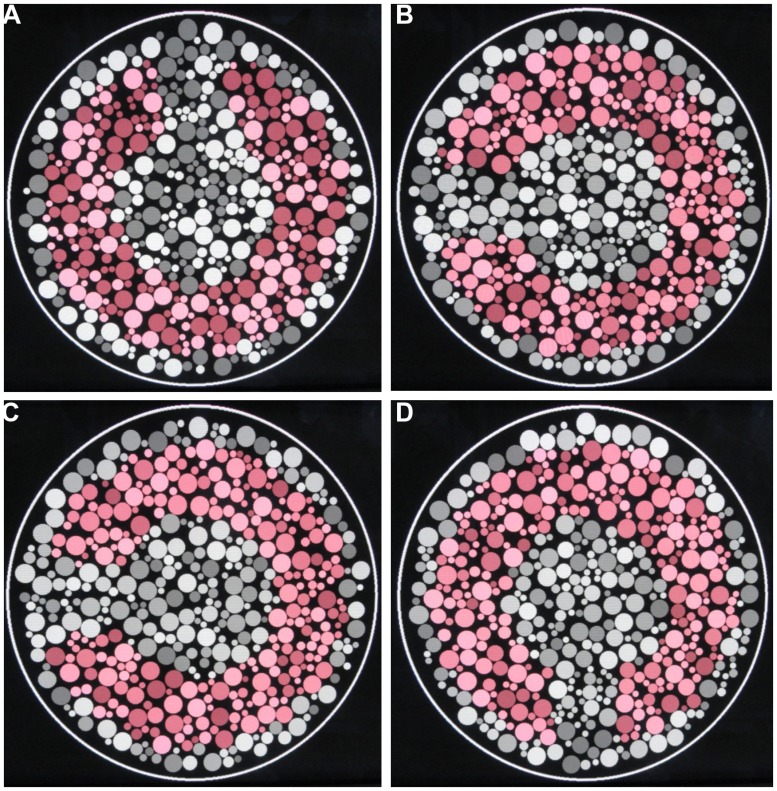
**Pseudoisochromatic stimuli used in this work. (A–D)** Four different categories of luminance levels in the luminance noise: 2, 6, 10, and 16 luminance levels.

The determination of the color discrimination ellipse was performed under eight different luminance step conditions: 2, 4, 6, 8, 10, 12, 14, and 16 (**Figure 1**). The luminance steps refer to the number of equally spaced luminance levels randomly distributed in the luminance noise range of the CCT stimulation. For all participants, eight different stimulus conditions were shown, each one with a different number of luminance levels in the luminance noise. For every stimulus condition, we estimated the color discrimination thresholds along eight different chromatic axes. The tests were performed twice with sections occurring separately in three different days. The stimulus conditions testing always started in the luminance step 2 and ended with luminance step 16.

### DATA ANALYSIS

An ellipse function was fitted to the eight color discrimination thresholds using the Khachiyan Ellipsoid Method (Khachiyan, 1979) implemented with Matlab R2013a routines (Mathworks, Natick, MA, USA). We calculated the area, major axis, and minor axis of the ellipses, and lengths of protan, deutan, and tritan vectors. These values were taken as parameters to compare color discrimination across different stimulus conditions. Subjects sequentially repeated the whole test twice and the results were averaged for each subject along the eight vectors. All the results were analyzed and presented as “grand means” for the group of nine subjects altogether.

For each subject, data of each parameter were divided by the maximum value to normalize the results across all testing conditions. The one-way ANOVA followed by Tukey *post hoc* test was used to compare the results (α = 0.05).

## RESULTS

**FIGURE 2:**
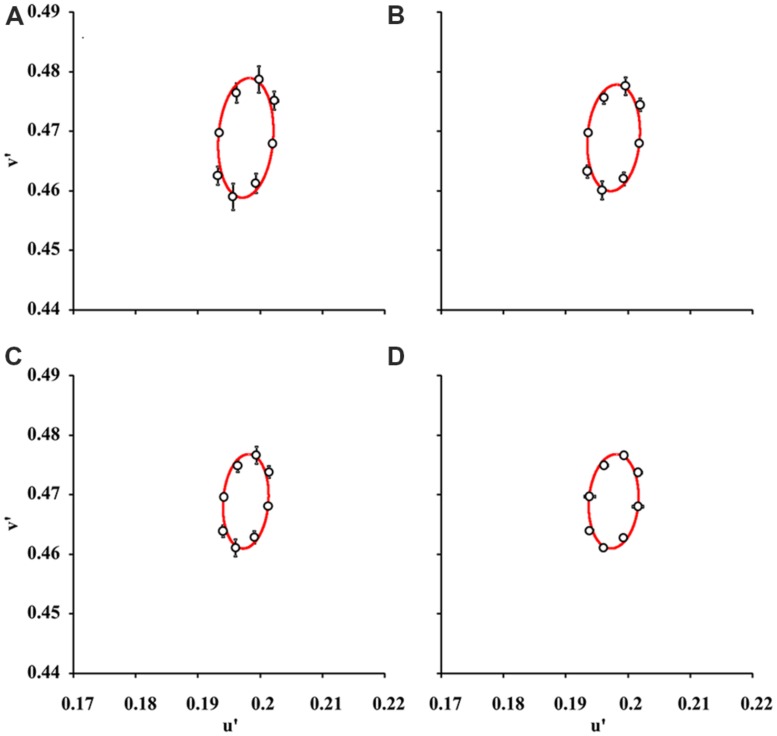
**Mean color discrimination ellipses for various degrees of luminance noise.** The data points and bars represent “grand means” and standard deviations for color discrimination thresholds in the CIE 1976 Color Space from nine subjects. Data points were fitted by ellipses estimated by using pseudoisochromatic stimuli with 2 **(A)**, 6 **(B)**, 10 **(C)**, and 16 **(D)** luminance levels in the luminance noise. The color discrimination thresholds obtained with two luminance levels in the luminance noise were higher than in any other conditions (*p* < 0.05, one-way ANOVA followed by Tukey *post hoc* test) and consequently the ellipse in **(A)** is larger than all other ellipses.

**Figure 2** shows the mean color discrimination ellipses in the CIE1976 Color Space for test conditions with 2, 6, 10, and 16 luminance levels in the luminance noise. Visual inspection reveals that the mean ellipse estimated with two luminance levels in the luminance noise (**Figure 2A**) had a larger area than the mean ellipses estimated with 6, 10, or 16 luminance levels on the luminance noise (**Figures 2B–D**).

**FIGURE 3:**
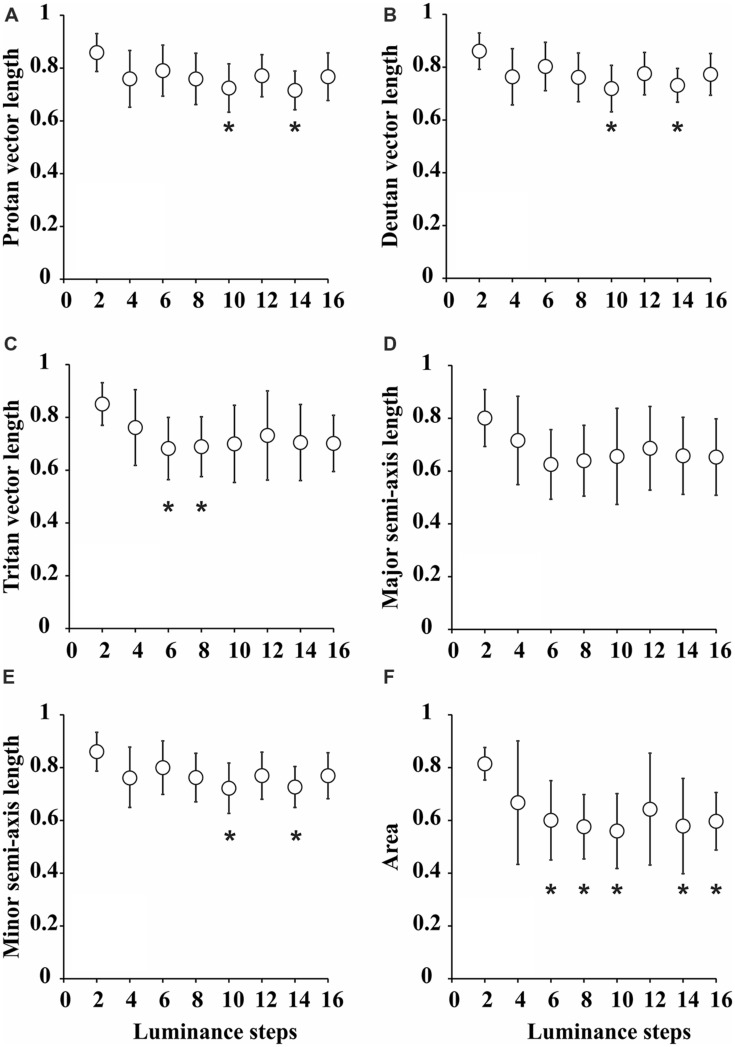
**Statistical comparisons between the parameters of color discrimination ellipses for various degrees of luminance noise. (A)** Protan vector length. **(B)** Deutan vector length. **(C)** Tritan vector length. **(D)** Major semi-axis length. **(E)** Minor semi-axis length. **(F)** Ellipse area. Values were “grand-means” for nine subjects that performed the tests twice and were averaged for each individual. Generally, all the parameters were larger for pseudoisochromatic tests performed with two luminance levels in the luminance noise, but only attained statistical significance level in a few comparisons for protan vector length [*F*(7,136) = 2.2, *p* < 0.05, η^2^ = 0.69], deutan vector length [*F*(7,136) = 2.3, *p* < 0.05, η^2^ = 0.7], tritan vector length [*F*(7,136) = 2.43, *p* < 0.05, η^2^ = 0.71], and minor semi-axis length [*F*(7,136) = 2.1, *p* < 0.05, η^2^ = 0.77]. For ellipses areas, most of comparisons were statistically significant. *p < 0.05, one-way ANOVA followed by Tukey *post hoc* test.

**Figure 3** summarizes all the statistical comparisons between the parameters for each test condition. All the parameters for ellipses obtained with two luminance levels in the luminance noise were larger than those for other seven combinations of luminance levels. However, statistical significance was reached only in few comparisons for one-dimensional parameters. There were no statistically significant differences for the comparisons between major semi-axis lengths estimated from the eight-luminance step conditions. The comparisons between the minor semi-axis lengths, protan vector lengths, deutan vector lengths, and tritan vector lengths resulted in statistically significant differences only in a few cases identified with asterisks in the plots.

We found that ellipse area was the best parameter that discriminated between different the test conditions. The ellipses for two luminance levels in the luminance noise had areas (0.81 ± 0.06) larger than for all other conditions and it was statistically significant larger [*F*(7,136) = 3.29, *p* < 0.05, η^2^ = 0.69] in the comparison with ellipses for 6 (0.60 ± 0.15), 8 (0.58 ± 0.12), 10 (0.56 ± 0.14), 14 (0.58 ± 0.18), and 16 (0.60 ± 0.11) luminance levels in the luminance noise.

## DISCUSSION

The luminance noise is an important feature of pseudoisochromatic tests. It is used to avoid borders and contrast between contiguous regions of the stimulus that would base the discrimination between target and background on cues other than chromatic differences. We found that decreasing the number of luminance levels composing the luminance noise impaired the color discrimination of trichromats, especially for very low numbers: two luminance levels resulted in larger ellipse areas; longer major semi-axes; longer minor semi-axes; and longer protan, deutan, and tritan vectors. That is, worse color discrimination thresholds were observed when compared to conditions with more luminance levels in the luminance noise.

The discrimination between target and background in pseudoisochromatic stimuli might be influenced by interaction between luminance and color information. Natural scenes are composed of both spatial and temporal mixture of color and luminance information, raising an interest in determining how these aspects are processed and discriminated within the visual system. Some authors suggested that the visual system performs an independent (orthogonal) and parallel processing of color and luminance (Livingstone and Hubel, 1987). This is supported by a scope of psychophysical and physiological data that showing distinct spatial and temporal properties of both the luminance and color channels (Wilson and Wilkinson, 2004; Solomon and Lennie, 2007). Others have suggested that the luminance and chromatic contribution for a perceptual task are summed at higher levels of the visual cortex (Switkes et al., 1988; Ingling and Grigsby, 1990; Logothetis et al., 1990; Gur and Akri, 1992).

The luminance and chromatic contrast processing might not be totally independent and they might in fact exert an influence upon each other. It is possible that independent processing of luminance and color information occurs only at the very early stages of the visual processing; however, it has been shown that many types of cells in the retina, lateral geniculate nucleus, and V1 respond to color and luminance contrast with varied degrees of sensitivities (Kaplan et al., 1988; Lee et al., 1989a,b, 1990, 2011; Johnson et al., 2001; Horwitz and Albright, 2005; Nassi and Callaway, 2009; Li et al., 2014).

The color information seems to potentiate the luminance contrast perception. Improvement in the spatial contrast sensitivity, wavelength discrimination, reaction times, and stereo-vision due the interaction of both chromatic and luminance information have been previously reported (Ueno and Swanson, 1989; Jordan et al., 1990; Logothetis et al., 1990; Gur and Akri, 1992). Gur and Akri (1992) investigated human contrast sensitivity that was estimated by luminance, chromatic, and compound luminance plus chromatic sinusoidal gratings. They observed that the luminance contrast sensitivity was enhanced by the addition of color information and *vice versa*. These investigators suggested that there was an additive mechanism that supported the enhancement of the contrast detection. Troscianko et al. (1996) conducted studies on color discrimination of two achromatopsic subjects using both static and dynamic (25 Hz) chromatic stimuli with luminance noise. They observed that subjects had the color discrimination impaired with static noise, but had normal performance with dynamic noise. These authors suggested that color discrimination estimated by static luminance would be relied by a conscious and color opponent mechanism reflecting probably the activity of both parvo- and magnocellular pathways. The color discrimination estimated using dynamic luminance noise would be performed by an unconscious and non-opponent mechanism that could be represented by the activation of the either the magnocellular or koniocellular pathways.

The current study, the number of possible combinations of luminance contrasts between two neighbor circular patches in the pseudoisochromatic stimuli varied according to the number of luminance steps. In the pseudoisochromatic stimuli of this study, two neighboring circular patches could vary from presenting the same luminance levels (0 contrast) to presenting the minimum and maximum luminance levels for that particular condition (highest contrast). In this study, the highest contrast was obtained between patches with 8 and 18 cd/m^2^ (Weber’s contrast = 0.55). The higher the number of luminance levels in the noise, the higher the number of possible luminance contrasts between two circular patches within the mosaic.

**FIGURE 4:**
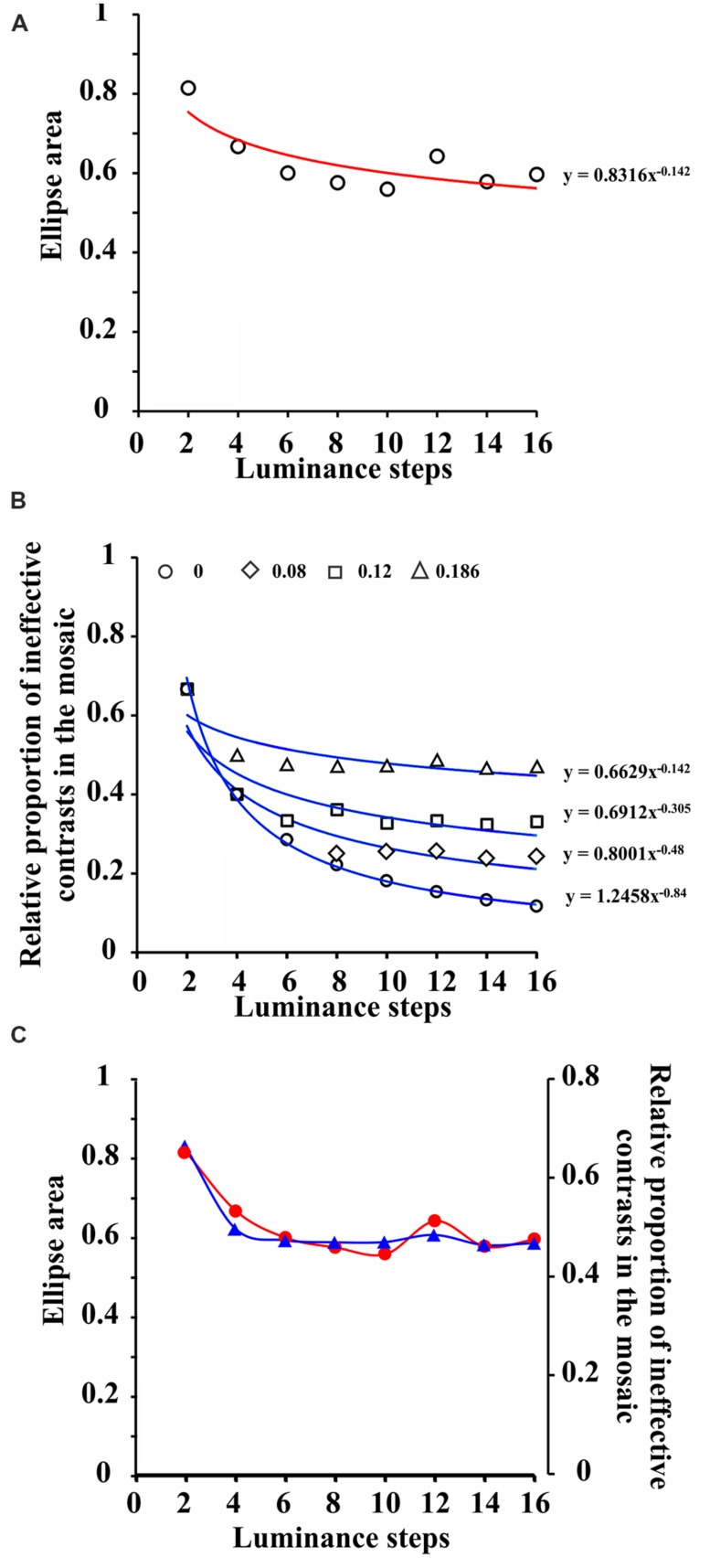
**Co-variation of the color discrimination ellipse areas and relative proportion of ineffective contrasts present in the mosaic. (A)** Area of color discrimination ellipses measured with pseudoisochromatic stimuli bearing progressive number of luminance steps in the luminance noise. Data points represent grand means (nine subjects, two measurements averaged for each individual) and were fitted with a power function. **(B)** Relative proportion of ineffective contrasts present in the mosaic of pseudoisochromatic stimuli as a function of number of luminance steps in the luminance noise. From top to bottom, the different groups of data points represent different values of Weber contrast threshold, and they were also fitted with power functions. Contrast threshold equal to 0.186 was fitted with a power function with the same exponent as the one for ellipses areas illustrated in **(A)**. **(C)** Data points for relative proportion of ineffective contrasts in the mosaic of pseudoisochromatic stimuli were vertically adjusted to fit the data points for color discrimination ellipse areas. The two sets of data points were connected with spline functions. (see text for further details).

It was hypothesized in the current study that not all possible luminance contrasts present within the mosaic could affect chromatic detection in the same amount and would explain the change in color discrimination threshold as a function of number of luminance steps in the luminance noise (**Figure 4A**). Low luminance contrasts do not have the same effect as high luminance contrasts to increase target chromatic integration since low contrasts may contribute little to the luminance noise. In order to find which luminance contrast could be the minimally effective contrast to modulate target integration, we estimated the relative proportion of ineffective luminance contrasts, present in each stimulus condition, assuming different contrast thresholds (**Figure 4B**). A Weber contrast threshold of 0.186 generates a relative proportion of ineffective contrasts, present in the mosaic, as a function of number of luminance steps in the luminance noise, which matches the color discrimination threshold as a function of number of luminance steps in the luminance noise (**Figure 4C**).

A Weber luminance contrast threshold equal to 0.186 is relatively high compared to the peak of human luminance contrast sensitivity, but it is compatible with a pathway of low luminance contrast sensitivity, such as the P cell pathway. P cell pathway could be an adequate candidate to integrate luminance contrast information and color contrast information in the perception of pseudoisochromatic stimuli, such as those used in the current study, since P cells are very sensitive to color contrast and relatively insensitive to luminance contrast (Kaplan and Shapley, 1986; Lee et al., 1989a,b, 1990, 2011).

## CONCLUSION

The CCT is a robust color discrimination test, since the values it provides for color discrimination thresholds are relatively insensitive to luminance noise. We suggest that stimulus conditions with six or more luminance levels would be more appropriate to test and characterize vision disabilities with pseudoisochromatic stimuli such the one used in the CCT. A pathway with high chromatic contrast sensitivity and low luminance contrast sensitivity seems to mediate the subject response to CCT. Pseudoisochromatic tests should have all the parameters fully described to allow for better interpretation of the results they provide and a more straightforward comparison of obtained results in different laboratories under different conditions.

## Conflict of Interest Statement

The authors declare that the research was conducted in the absence of any commercial or financial relationships that could be construed as a potential conflict of interest.
